# Increased risk of injury and adult attention deficit hyperactivity disorder and effects of pharmacotherapy: a nationwide longitudinal cohort study in South Korea

**DOI:** 10.3389/fpsyt.2024.1453100

**Published:** 2024-12-10

**Authors:** Jaeun Ahn, Jae-won Shin, Haeyong Park, Joong-Won Ha

**Affiliations:** ^1^ Department of Psychiatry, National Health Insurance Service Ilsan Hospital, Goyang, Republic of Korea; ^2^ Institute of Behavioral Science in Medicine, Yonsei University College of Medicine, Seoul, Republic of Korea; ^3^ Department of Orthopedic Surgery, Yonsei University College of Medicine, Seoul, Republic of Korea; ^4^ Research and Analysis Team, National Health Insurance Service Ilsan Hospital, Goyang, Gyeonggi, Republic of Korea; ^5^ Department of Orthopedic Surgery, National Health Insurance Service Ilsan Hospital, Goyang, Republic of Korea

**Keywords:** attention deficit hyperactivity disorder, ADHD, injury, methylphenidate, atomoxetine

## Abstract

Children and adolescents with attention deficit hyperactivity disorder (ADHD) are at an increased risk of accidents and injuries, and ADHD medication has been shown to mitigate this risk in these populations. However, the injury risk and the influence of ADHD medication in adults with ADHD remain unclear. This study aimed to investigate the injury risk in adults with ADHD and assess the impact of ADHD medication on this risk. Using a nationwide health claims database, we identified 9,417 adult patients with ADHD aged 19–44 years between 2017 and 2018. A retrospective propensity score-matched case-control study was conducted to examine the association between adult ADHD and injury risk across various categories. The effects of two commonly prescribed ADHD medications, methylphenidate and atomoxetine, were evaluated using a Cox proportional hazards model. The results showed that adults with ADHD had an increased risk of sustaining various types of injuries. Methylphenidate demonstrated a protective effect against injury, which persisted after adjusting for potential confounding factors. Similarly, atomoxetine significantly reduced the injury risk. These findings underscore the importance of injury prevention strategies in adults with ADHD and highlight the substantial health benefits of ADHD medications in this population.

## 1 Introduction

Attention deficit hyperactivity disorder (ADHD) is a prevalent neurodevelopmental disorder characterized by significant difficulties in attention, hyperactivity, impulsiveness, or a combination of these traits (1). ADHD is a neurodevelopmental disorder that often begins in childhood and persists into adulthood in approximately 65% of those diagnosed during childhood, according to a previous meta-analysis (2). Additionally, the prevalence of ADHD in adults is estimated to be around 4-5% globally (3, 4). Assessing the injury risk in adults with ADHD is crucial, as core symptoms such as inattention, impulsivity, and hyperactivity can impair risk assessment and response, increasing their susceptibility to accidents and injuries (5).

Evidence suggests a potential link between ADHD and increased risk of injury. Psychosocial characteristics such as impulsivity, poor judgment, and a tendency to seek immediate gratification may explain the elevated risk of injuries (6). For example, Farmer and Peterson (7) reported that children with ADHD, while capable of identifying hazards, anticipated relatively low severity in the consequences of their behavior. Furthermore, a recent systematic review and meta-analysis demonstrated a 1.3- to 2.2-fold increase in the prevalence of fractures among children with ADHD compared with those without ADHD (8). A large matched-cohort study indicated that children with ADHD were 1.2 times more likely to experience fractures than their counterparts without ADHD, with an even greater risk of developing multiple fractures (9).

Treatment options for ADHD include pharmacological and non-pharmacological approaches. As with childhood and adolescent ADHD, pharmacotherapy for adult ADHD is highly effective (10) and is widely endorsed as the first-line treatment by the National Institute for Health and Care Excellence (NICE) (11). The medications approved by the Korean Food and Drug Administration (KFDA) for adult ADHD include psychostimulant drugs (e.g., methylphenidate) and non-psychostimulant drugs (e.g., atomoxetine and the α2 adrenoceptor agonist, clonidine) (12). A large matched-cohort study suggested that pharmacological treatment for children with ADHD reduces the fracture risk (9); however, research on the effects of such treatment in adults remains limited (5).

Given the potentially increased risk of injury in adults with ADHD, it is essential to investigate the risk of injury in this population and assess the impact of pharmacotherapy. However, research on adults with ADHD is scarce because ADHD has historically been viewed as a childhood disorder. This study aimed to address two key aspects: first, whether there is a difference in the incidence of injury-related morbidity in adults with ADHD compared with those without, and second, whether pharmacological treatment for ADHD reduces the morbidity of injury in adults with ADHD.

## 2 Materials and methods

### 2.1 Data sources

We utilized a comprehensive nationwide database provided by the National Health Insurance Service (NHIS), the single provider of universal healthcare coverage in South Korea (13). The NHIS covers the entire South Korean population, including individuals supported by the Medical Aid Program for those who cannot afford NHIS coverage. This ensures that all citizens are included under either the NHIS or the Medical Aid Program. The NHIS database contains detailed sociodemographic information, insurance status, and extensive medical records, including diagnoses classified by the International Classification of Diseases, 10th Revision (ICD-10), as well as records of medical services, procedures, and associated costs claimed by healthcare providers. It also includes prescription records and health screening results, offering a comprehensive overview of patient care. Additional details of the NHIS database have been previously described (13, 14). This study complied with the Declaration of Helsinki, and the study protocol was approved by the Institutional Review Board of the National Health Insurance Service Ilsan Hospital (NHIMC 2022-02-004). Written informed consent was waived because the study was based on anonymous administrative data.

### 2.2 Study population selection

Individuals with ADHD were identified using the ICD-10 diagnosis code (F90). A total of 9417 adults aged 19–44 years who were newly diagnosed with ADHD between January 1, 2017 and December 31, 2018, were enrolled in the study cohort. To ensure diagnostic reliability and to clearly differentiate the ADHD group from the control group, we included only participants with at least three separate ADHD diagnoses, either in an outpatient clinic or in the Department of Psychiatry. This approach reduces the likelihood of false-positive diagnoses, aligning with methodologies used in previous epidemiologic studies (15–17), and accounts for the diagnostic complexity of adult ADHD, where incomplete childhood memories may hinder accurate self-reporting and retrospective assessments (18, 19).

**Figure 1:**
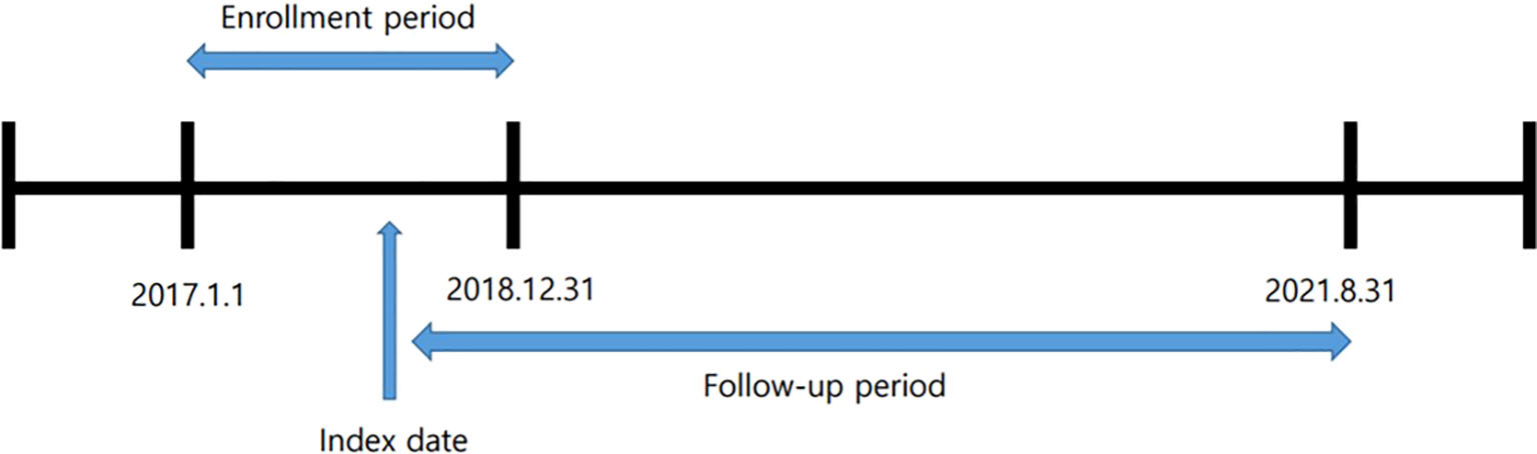
The study design for eligible patient selection.

The ‘index date’ was defined as the date when participants were first enrolled in the cohort after receiving an ADHD diagnosis on at least three separate occasions. Participants were followed from the index date until August 31, 2021. This follow-up period was used to assess the association between ADHD diagnosis and injury risk (**Figure 1**). For each adult ADHD case, a matched control group was randomly selected from the NHIS database using propensity score matching to correct for the potential confounding effect of baseline characteristic differences between the adult ADHD and control groups (20, 21).

The propensity scores were estimated using a multivariate logistic regression model. The covariates in the model included age, sex, household income, region of residence, and insurance type. Based on propensity scores, we matched participants without adult ADHD to those with adult ADHD in a 5:1 ratio using a local optimal algorithm. This approach enabled us to determine whether adult ADHD was an independent outcome predictor.

### 2.3 Outcomes

Injuries (fractures, dislocations, sprains and strains, intracranial/internal injuries, open wounds, injury to blood vessels, superficial injuries/contusions, crushing injuries, burns, injury to nerves and spinal cord, poisoning, and other specified and unspecified injuries) were defined using ICD-10 codes (**Supplementary Table 1**) Injuries were categorized into three groups: total, mild, and severe. The mild injury group comprised patients diagnosed using this code in outpatient clinics or emergency rooms. In contrast, the severe injury group comprised patients admitted to the hospital with this code.

Comorbid psychiatric disorders (mood disorder, anxiety disorder, stress-related disorder, and substance-related disorder) were defined using the ICD-10 codes (**Supplementary Table 2**).

### 2.4 Other variables

To adjust for the potential effects of comorbidities such as cardiovascular disease, malignancies, and diabetes mellitus, we used Quan’s updated Charlson Comorbidity Index algorithm (22).

### 2.5 Statistical analysis

The baseline demographics and comorbidities of participants with and without ADHD were characterized using descriptive statistics and compared using absolute standardized difference. Multivariable Cox proportional hazard regression models were used to explore the association between ADHD and risk of injury. The main explanatory variable was the absence of ADHD. The models were adjusted for baseline age, sex, income, region of residence, and insurance type with the potential to cause injury-related diseases. We applied Cox regression models after stratifying the participants with and without ADHD. The proportional hazard assumption was tested using Schoenfeld residuals.

To determine whether ADHD medication reduces the occurrence of injury, we compared patients with ADHD who were prescribed medication for more than three months with those who were not. We selected methylphenidate and atomoxetine, both widely prescribed and approved by the Korean Food and Drug Administration (KFDA) for adult ADHD treatment. Methylphenidate, a stimulant, reduces impulsivity and may lower injury risk, while Atomoxetine, a norepinephrine reuptake inhibitor, offers a non-stimulant alternative with proven efficacy in managing ADHD symptoms (10–12).

We conducted a sensitivity analysis to assess the effect of ADHD medications (methylphenidate and atomoxetine) on reducing the occurrence of injury. In the sensitivity analysis, we categorized the participants into four groups based on the duration of ADHD medication: those who received no medication, those who received medication for <3 months, those who received medication for 3–6 months, and those who received medication for more than six months.

Data from the descriptive analyses were summarized using means, medians, or numbers (proportions), as appropriate, and compared using independent sample *t*-tests or chi-square tests. All the analyses were performed using SAS version 9.4 (SAS Institute Inc., Cary, NC, USA). A *P* value <.05 was used as the threshold for statistical significance for any tests.

### 2.6 Data availability

The datasets analyzed in this study are not available to the public because of NHIS restrictions and are stored on separate servers managed by the NHIS. The NHIS requires an interested party to access data. Applications were submitted online (nhiss.nhis.or.kr). They required a study proposal and ethical approval from the Institutional Review Board.

## 3 Results

### 3.1 Clinical characteristics of the study population

The study population consisted of 56,502 individuals, including those diagnosed with adult ADHD and a control group. Detailed demographic information is presented in **Table 1**. The largest age group, 25–29 years, accounted for 27.6% of participants. The second-largest age group comprised individuals aged 19–24, accounting for 25.9% of participants. Furthermore, the study included individuals aged 30–34, 35–39, and 40–44 years, contributing varying proportions to the overall population. The gender distribution revealed that 62.3% of the participants were male and 37.8% were female, indicating a balanced representation of both genders. The participants’ household income levels were categorized into quintiles (Q1, Q2, Q3, Q4, and Q5), from lowest to highest. The highest income quintile (Q5) comprised the largest proportion of participants, accounting for 31.12% of the study population. Additionally, the distribution across income quintiles was relatively uniform, with each quintile contributing significantly to the overall study cohort. The participants’ residential areas were classified into metropolitan, urban, and rural. Most participants resided in metropolitan areas, constituting 53.4% of the study population. Urban areas were home to 43.2% of participants, whereas 3.1% lived in rural areas.

**Table 1:** Baseline characteristics of participants.

	Total	Adult ADHD	Control
	N = 56,502	N = 9,417	N = 47,085
Age, years			
19–24	14,646(25.9)	2,441	12,205
25–29	15,600(27.6)	2,600	13,000
30–34	10,290(18.2)	1,715	8,575
35–39	5,730(10.1)	955	4,775
40–44	10,236(18.1)	1,706	8,530
Sex			
Male	35172(62.3)	5,862	29,310
Female	21330(37.8)	3,555	17,775
Household income^*^			
Q1, lowest	9,528(16.9)	1,588	7,940
Q2	9,450(16.7)	1,575	7,875
Q3	10,086(17.9)	1,681	8,405
Q4	9,852(17.4)	1,642	8,210
Q5, highest	17,586(31.12)	2,931	14,655
Residential area			
Metropolitan	30,204(53.4)	5,034	25,170
Urban	24,450(43.2)	4,075	20,375
Rural	1,794(3.1)	299	1,495

Values are presented as the median [interquartile range] or number (%).

^*^Categorized based on quartiles in the entire Korean population.

Among the study participants, 961 adults with ADHD received atomoxetine for >three months, and 995 adults with ADHD received atomoxetine for <3 months. Additionally, 7,461 adults with ADHD did not receive atomoxetine treatment. In the case of methylphenidate, 5,973 adults with ADHD received it for >3 months, 2,579 for <3 months, and 1,860 adults with ADHD did not receive it.

### 3.2 Association between adult ADHD and risk of injury

In our study, we observed that adults with ADHD had a substantially higher risk of injury than their non-ADHD counterparts. Among adults with ADHD, 39.51% reported total injuries, which was significantly higher than the 23.96% reported in the non-ADHD group. Similarly, the incidence of severe injuries was higher among adults with ADHD (12.36%) than among those without it (5.21%). Mild injuries were more frequent among adults with ADHD, with a prevalence of 33.88%, which significantly exceeded the prevalence of 20.64% observed in the non-ADHD group.

**Figure 2:**
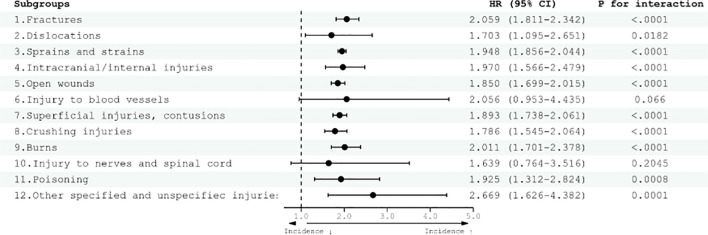
Risk of total injury diseases according to adult ADHD diagnosis. HRs were adjusted for age, sex, household income, and residential area. CI, confidence interval; HR, hazard ratio.

In the context of adult ADHD, when compared with the control group, an elevated risk of total injury was observed (**Figure 2**). The hazard ratios (HR) for various injury categories were as follows: fractures (HR 2.059, 95% confidence interval (CI) 1.811–2.342), dislocations (HR 1.703, 95% CI 1.095–2.651), sprains and strains (HR 1.948, 95% CI 1.856–2.044), intracranial/internal injuries (HR 1.970, 95% CI 1.566–2.479), open wounds (HR 1.850, 95% CI 1.699–2.015), superficial injuries and contusions (HR 1.893, 95% CI 1.738–2.061), crushing injuries (HR 1.786, 95% CI 1.545–2.064), burns (HR 2.011, 95% CI 1.701–2.378), poisoning (HR 1.925, 95% CI 1.312–2.824), and other specified and unspecified injuries (HR 2.669, 95% CI 1.626–4.382).

**Figure 3:**
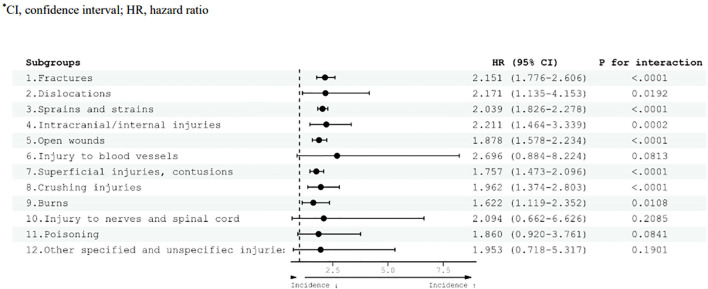
Risk of severe injury diseases according to adult ADHD diagnosis. HRs were adjusted for age, sex, household income, and residential area. CI, confidence interval; HR, hazard ratio.

In adults with ADHD, a confirmed increase in the risk of severe injury was observed compared with the control group (**Figure 3**). The HR for the various categories of severe injuries demonstrated this heightened risk. For example, fractures exhibited an HR of 2.151 (95% CI 1.776–2.606), indicating a substantially elevated risk. Similarly, dislocations showed an HR of 2.171 (95% CI, 1.135–4.153), highlighting a significant increase in risk within this category. Other severe injury categories displayed HR, such as sprains and strains with an HR of 2.039 (95% CI 1.826–2.278), intracranial/internal injuries with an HR of 2.211 (95% CI 1.464–3.339), and open wounds with an HR of 1.878 (95% CI 1.578–2.234). Superficial injuries and contusions exhibited an elevated risk, with an HR of 1.757 (95% CI, 1.473–2.096); crushing injuries had an HR of 1.962 (95% CI 1.374–2.803); and burns had an HR of 1.622 (95% CI 1.119–2.352).

**Figure 4:**
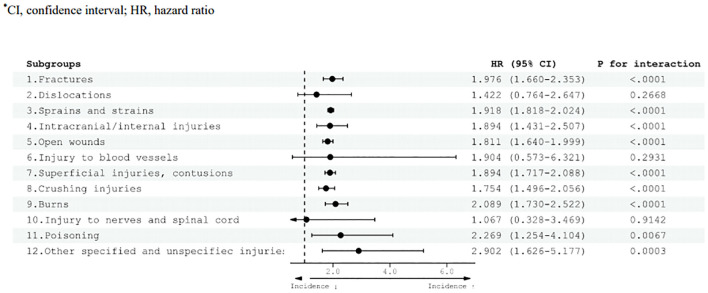
Risk of mild injury diseases according to adult ADHD diagnosis. HRs were adjusted for age, sex, household income, and residential area. CI, confidence interval; HR, hazard ratio.

In adults with ADHD, an elevated risk of mild injury was observed compared with the control group (**Figure 4**). Specifically, the HR for various injury categories were as follows: fractures (HR 1.976, 95% CI 1.660–2.353), sprains and strains (HR 1.918, 95% CI 1.818–2.024), intracranial/internal injuries (HR 1.894, 95% CI 1.431–2.507), open wounds (HR 1.811, 95% CI 1.640–1.999), superficial injuries and contusions (HR 1.894, 95% CI 1.717–2.088), crushing injuries (HR 1.754, 95% CI 1.496–2.056), burns (HR 2.089, 95% CI 1.730–2.522), poisoning (HR 2.269, 95% CI 1.254–4.104), and other specified and unspecified injuries (HR 2.902, 95% CI 1.626–5.177).

### 3.3 Effects of pharmacological treatment for adult ADHD and risk of injury

**Figure 5:**
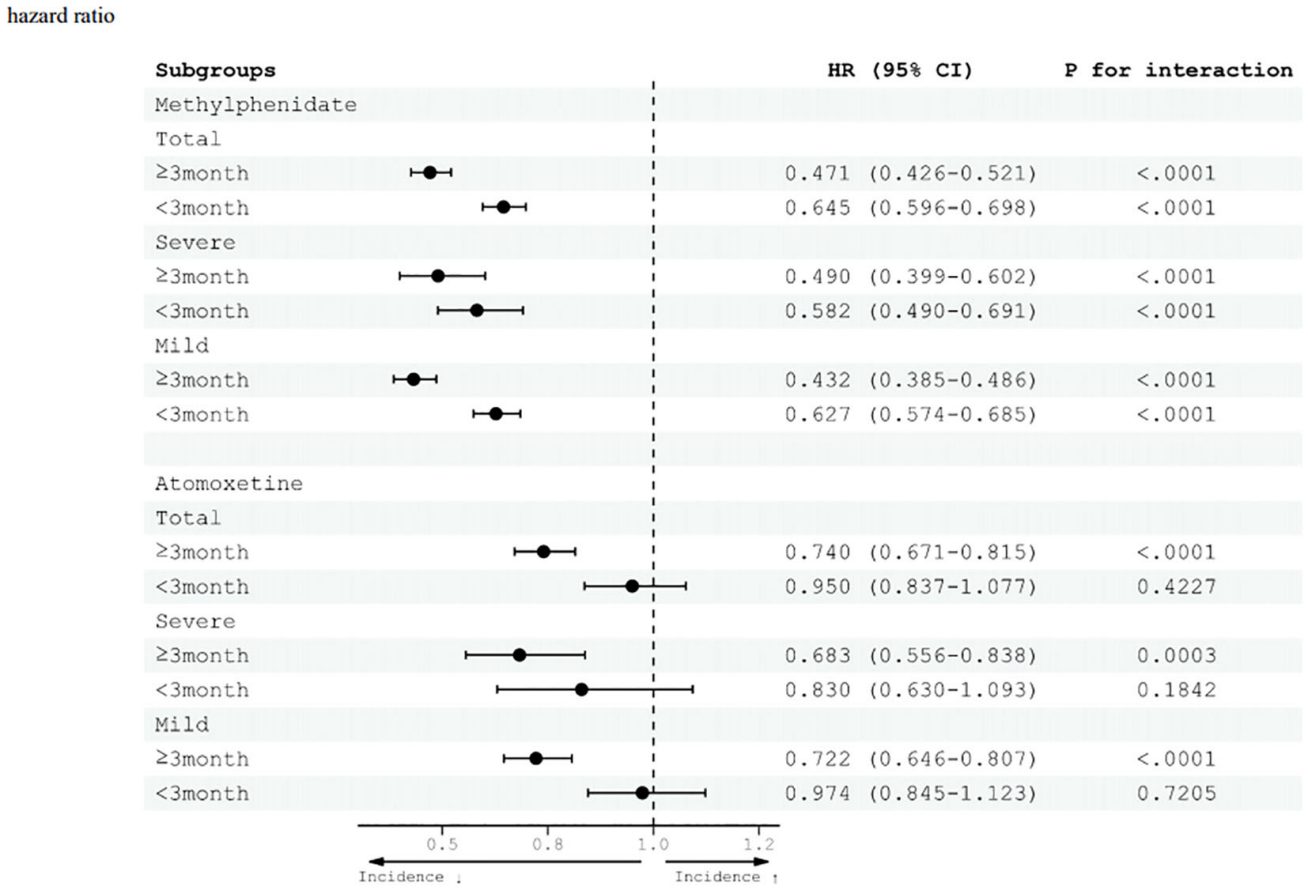
Effects of pharmacological treatment (methylphenidate and atomoxetine) for adult ADHD and risk of injury diseases. CI, confidence interval; HR, hazard ratio.

In both methylphenidate treatment groups, those who received medication for more than three months and those who received it for less than three months showed a significant reduction in the risk of total, mild, and severe injuries (**Figure 5**). In the group receiving methylphenidate for >3 months, the risk of total and severe injuries decreased significantly, with HR values of 0.471 (95% CI 0.426–0.521) and 0.490 (95% CI 0.399–0.602), respectively. Additionally, the risk of mild injury decreased, with an HR of 0.432 (95% CI 0.385–0.486). Furthermore, for patients administered methylphenidate for <3 months, there was a notable reduction in the risk of injury compared with those who did not receive the medication. The risk of total, severe, and mild injuries decreased, with HR values of 0.645 (95% CI, 0.596–0.698), 0.582 (95% CI, 0.582–0.691), and 0.627 (95% CI, 0.574–0.685), respectively.

In the atomoxetine treatment group, a decrease in the risk of injury was observed only when the drug was administered for >3 months. In the group receiving atomoxetine for <3 months, there was no significant difference in the risk of injury compared with the group not receiving atomoxetine. Among those who took atomoxetine for ≥3 months, the risk of total injury decreased with an HR of 0.740 (95% CI 0.671–0.815) compared with the group not taking the medication. The risks of severe and mild injuries decreased, with HR values of 0.683 (95% CI 0.556–0.838) and 0.722 (95% CI 0.646–0.807), respectively.

### 3.4 Sensitivity analyses

To strengthen the validity of the study outcomes, a sensitivity analysis was conducted to investigate the effect of the duration of ADHD medication on injuries. The findings of the sensitivity analysis are consistent with those of the primary analysis. In the group receiving methylphenidate for >6 months, the risk of total injury decreased significantly, with an HR of 0.483 (95% CI 0.435–0.535). Additionally, in the group receiving methylphenidate for >3 months, the risk of total injury decreased significantly, with an HR of 0.661 (95% CI 0.609–0.717). Furthermore, even in the group undergoing drug therapy for <3 months, the risk of total injury decreased significantly with an HR of 0.665 (95% CI 0.596–0.698).

In the atomoxetine treatment group, a reduction in the risk of injury was observed when the drug was administered for ≥3 months. In the group receiving atomoxetine for >6 months, the risk of total injury decreased significantly, with an HR of 0.790 (95% CI 0.704–0.886). Additionally, in the group receiving atomoxetine for ≥3 months, the risk of total injury decreased significantly, with an HR of 0.740 (95% CI 0.671–0.815).

## 4 Discussion

ADHD, a neurodevelopmental disorder often associated with childhood, is increasingly recognized for its persistence and implications in adulthood (23). This comprehensive longitudinal cohort study, conducted nationwide in South Korea, examined the vulnerability of adults with ADHD to injuries and explored the potential risk mitigation offered by pharmacological interventions. Based on our study, the risk of injury increases as a person suffers from adult ADHD and peaks in young adults. In this study, we identified the age groups with a particularly elevated risk of injury. From our study results, there seemed to be a cluster around young adults (18–25 years old) where the injury risk peaked. Patients with ADHD aged 18–25 years who present to the hospital/emergency room should be screened to reduce the risk of further accidents and injuries.

Our findings confirm that adults with ADHD exhibit significantly augmented susceptibility to injuries compared with their non-ADHD counterparts. This heightened predisposition transcends the boundaries of injury severity, encompassing both severe and minor injuries and specific injury subcategories. The escalated proclivity toward injuries in the ADHD-afflicted population traverses the spectrum of adult age groups, indicating that this association is not circumscribed to any particular phase of adulthood.

Our study is the first to investigate the relationship between adult ADHD and injury risk in a South Korean population, and it benefits from the analysis of a large sample size, which strengthens the generalizability and robustness of our findings. With over 9,000 adults with ADHD included in the study, we provide insights based on one of the largest datasets focused on adult ADHD and injury risk in this region. Furthermore, unlike many previous studies that emphasize children or adolescents with ADHD (5), our research centers specifically on adults, a group that continues to face significant challenges related to injury risks.

One noteworthy finding of our study was the association between the pharmacological treatment of ADHD and a reduction in the risk of injury. This observation aligns with recent research suggesting the potential protective effect of ADHD medications against injury (9). The precise mechanisms underlying this risk reduction warrant further investigation; however, several hypotheses can be considered. Although the precise underlying mechanisms require further elucidation, several plausible hypotheses have been proposed.

Our study suggests that stimulant medications, particularly methylphenidate, may play a significant role in reducing the risk of injury among adults with ADHD. These medications have been acknowledged for their ability to enhance attentional faculties and improve impulse control, which are core deficits in individuals with ADHD (2). Augmented attentional focus and improved impulse regulation may lead to improved behavioral safety, heightened risk appraisal, and diminished proclivity toward accidents and injuries (24). In addition, ameliorating ADHD symptoms through pharmacological intervention may foster overall functional enhancement and bolster quality of life. Although the exact mechanisms remain unclear, our findings also suggest that stimulant medications, particularly methylphenidate, may reduce the risk of injury in adults with ADHD by mitigating core symptoms like inattention and impulsivity, which are linked to accidents. Patients undergoing treatment may be better equipped to navigate daily responsibilities and negotiate intricate life scenarios, thereby reducing their vulnerability to accidents.

Additionally, to the best of our knowledge, there are no studies that have specifically investigated the preventive effects of different ADHD medications (stimulants and non-stimulants) on accident and injury risk across different age groups (5). Our study is unique in its detailed analysis of the effects of ADHD pharmacotherapy, not only comparing stimulants and non-stimulants but also stratifying patients by the duration of treatment. By examining patients who were treated with methylphenidate or atomoxetine for varying lengths of time, we provide nuanced insights into how prolonged versus short-term treatment influences injury risk. Our findings suggest that longer durations of pharmacotherapy are associated with a greater reduction in injury risk, setting this study apart from others that have primarily focused on the general benefits of ADHD medications without accounting for both treatment type and duration.

Our findings also highlight the potential benefits of implementing ADHD screening, particularly for patients who present with recurrent or unexplained injuries. Core symptoms of ADHD, such as inattention, impulsivity, and hyperactivity, can impair an individual’s ability to accurately assess risks and respond to dangerous situations, thereby increasing their susceptibility to accidents (23, 25–28). Early detection and intervention could not only mitigate the risk of future injuries but also improve patient outcomes and reduce the broader healthcare burden associated with untreated ADHD in adulthood. Expanding ADHD screening to include emergency and trauma settings may offer clinicians the opportunity to identify and address this underlying condition in high-risk patients, potentially reducing injury rates and enhancing overall patient safety.

Moreover, our findings underscore the importance of the early detection and accurate diagnosis of ADHD in adults. Timely intervention and commencement of suitable therapeutic modalities may protect against the elevated injury risk associated with untreated or undiagnosed ADHD. Clinicians should be informed about the increased risk of accidents and injuries among adults with ADHD. These measures offer substantial advantages for individuals and society through injury prevention and healthcare cost reductions.

The strengths of our study include the formidable dataset derived from a vast and heterogeneous cohort, meticulous categorization of diverse injury types, and longitudinal study design. Despite the strengths of this study, several limitations should be acknowledged. First, we relied on administrative data, which may have inadvertently overlooked nuanced clinical variables, including ADHD severity and treatment adherence. Second, we were unable to adjust for the potential influence of concomitant medications taken for other conditions. The use of additional medications may affect injury risk in adults with ADHD, and future studies should consider this factor when analyzing injury outcomes. Third, while osteoporosis and other diseases affecting bone health are known to influence injury risk, particularly fractures, the relatively young age of our study population (19–44 years) suggests a low prevalence of osteoporosis. Previous research indicates that osteoporosis is uncommon in this age group (29). Nonetheless, the exclusion of bone health conditions remains a limitation, and future research with older populations should explore this relationship more thoroughly. Fourth, One important limitation of this study is the inability to adjust for specific types of accidents, such as traffic accidents, due to the limitations of the National Health Insurance Service (NHIS) database (13). The database provides injury data based on ICD-10 codes, but it lacks detailed information regarding the context or cause of the accidents (e.g., whether the injury occurred due to a traffic accident, fall, or other incident). As a result, we were unable to fully explore the potential confounding effects of different accident types on the injury outcomes in adults with ADHD. Future studies with more detailed data on accident contexts are needed to address this limitation. Fifth, an important limitation of this study is the inability to analyze the effects of non-pharmacological interventions such as psychoeducation, cognitive-behavioral therapy, or psychotherapy, as these are not systematically recorded in the NHIS database. Therefore, future research should investigate both pharmacological and non-pharmacological treatments to better understand their combined impact on injury risk in individuals with adult ADHD.

In summary, our investigation provides compelling evidence that adults with ADHD exhibit a heightened proclivity toward injuries, spanning diverse injury typologies and severity gradations. This underscores the potential merits of pharmacological interventions in ameliorating this risk. These findings highlight the need for the early identification of ADHD in adults, the timely institution of accurate diagnoses, and the development of efficacious treatment strategies to curtail the augmented risk of injuries encountered by this demographic. Further research is needed to unravel the intricate mechanisms of these phenomena and delineate optimal paradigms for injury prevention in adults with ADHD.

## 5 Conclusions

Our nationwide longitudinal cohort study in South Korea revealed that adults with ADHD have a substantially increased risk of various types and severities of injuries. This risk is particularly pronounced in adults aged 18–25 years. Our findings suggest that pharmacological interventions, including psychostimulants, may mitigate this risk. Early diagnosis, timely treatment, and enhanced screening for ADHD in young adults can contribute significantly to injury prevention and public safety. Our research underscores the importance of recognizing ADHD as a serious concern in adulthood and highlights the potential benefits of proactive interventions in reducing injury-related healthcare costs and improving the well-being of affected individuals and society.

## Data Availability

The datasets presented in this article are not readily available because of NHIS restrictions and are stored on separate servers managed by the NHIS. The NHIS requires an interested party to access data. Applications were submitted online (nhiss.nhis.or.kr). They required a study proposal and ethical approval from the Institutional Review Board. Requests to access the datasets should be directed to JA, jaeun.ahn87@gmail.com.
