# Supplementary material for: Increased risk of injury and adult attention deficit hyperactivity disorder and effects of pharmacotherapy: a nationwide longitudinal cohort study in South Korea
Source: Front Psychiatry. 2024 Dec 10;15:1453100. doi: 10.3389/fpsyt.2024.1453100 (PMC11666558; doi:10.3389/fpsyt.2024.1453100)
Supplement: Supplementary file 1 [file DataSheet1.docx]

Supplementary Material

## Supplementary Figures


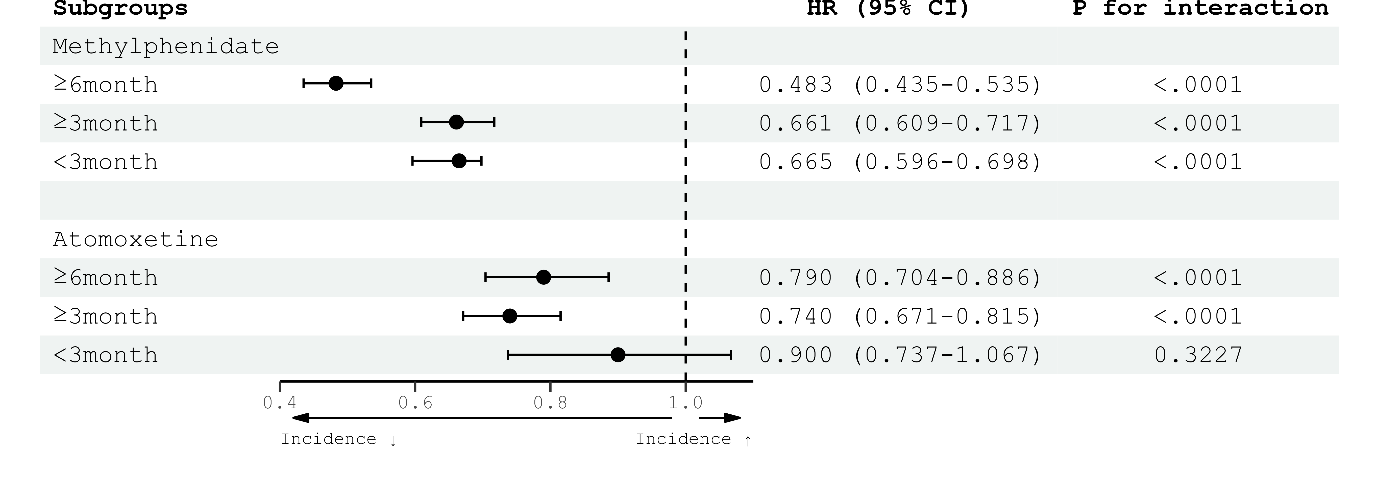


**Supplementary Figure 1.** Effects of pharmacological treatment duration (methylphenidate and atomoxetine) on adult ADHD and risk of injury diseases. *CI, confidence interval; HR, hazard ratio

## Supplementary Tables

**Supplement Table 1.** ICD-10 codes for injury diseases

| Injury diseases | ICD-10 codes |
| --- | --- |
| Fractures | -S02.0~9 Fracture of skull and facial bones  -S12.0~9 Fracture of neck  -S22.0~9 Fracture of rib(s), sternum and thoracic spine  -S32.0~8 Fracture of lumbar spine and pelvis  -S42.0~9 Fracture of shoulder and upper arm  -S52.0~9 Fracture of forearm  -S62.0~8 Fracture of wrist and hand level  -S72.0~9 Fracture of femur  -S82.0~9 Fracture of lower leg, including ankle  -S92.0~9 Fracture of foot, except ankle  -T02.0~9 Fractures involving multiple body regions  -T08 Fracture of spine, level unspecified  -T10 Fracture of upper limb, level unspecified  -T12 Fracture of lower limb, level unspecified  -T14.2 Fracture of unspecified body region |
| Dislocations | -S03.0~3 Dislocation, sprain and strain of joints and ligament of head  -S13.0~3 Dislocation, sprain and strain of joints and ligaments at neck level  -S23.0~2 Dislocation, sprain and strain of joints and ligaments of thorax  -S33.0~3 Dislocation, sprain and strain of joints and ligaments of lumbar spine and pelvis  -S43.0~3 Dislocation, sprain and strain of joints and ligaments of shoulder girdle  -S53.0~1 Dislocation, sprain and strain of joints and ligaments of elbow  -S63.0~3 Dislocation, sprain and strain of joints and ligaments at wrist and hand level  -S73.0 Dislocation, sprain and strain of joint and ligaments of hip  -S83.0~1 Dislocation, sprain and strain of joints and ligaments of knee  -S93.0~1 Dislocation, sprain and strain of joints and ligaments at ankle and foot level  -T03.0~9 Dislocation, sprains and strains involving multiple body regions  -T14.3 Dislocation, sprain and strain of unspecified body region |
| Sprains and strains | -S03.4~5 Dislocation, sprain and strain of joints and ligament of head  -S09.1 Injury of muscle and tendon of head  -S13.4~6 Dislocation, sprain and strain of joints and ligaments at neck level  -S16 Injury of muscle and tendon at neck level  -S23.3~5 Dislocation, sprain and strain of joints and ligaments of thorax  -S33.4~7 Dislocation, sprain and strain of joints and ligaments of lumbar spine and pelvis  -S43.4~7 Dislocation, sprain and strain of joints and ligaments of shoulder girdle  -S46.0~9 Injury of muscle and tendon at shoulder and upper arm level  -S53.2~4 Dislocation, sprain and strain of joints and ligaments of elbow  -S56.0~9 Injury of muscle and tendon at forearm level  -S63.4~7 Dislocation, sprain and strain of joints and ligaments at wrist and hand level  -S66.0~9 Injury of muscle and tendon at wrist and hand level  -S73.1 Dislocation, sprain and strain of joint and ligaments of hip  -S76.0~7 Injury of muscle and tendon at hip and thigh level  -S83.2~7 Dislocation, sprain and strain of joints and ligaments of knee  -S86.0~9 Injury of muscle and tendon at lower leg level  -S93.2~6 Dislocation, sprain and strain of joints and ligaments at ankle and foot level  -S96.0~9 Injury of muscle and tendon at ankle and foot level  -T03.0~9 Dislocation, sprains and strains involving multiple body regions  -T06.4 Injuries of muscles and tendons involving multiple body regions  -T09.2 Dislocation, sprain and strain of unspecified joint and ligament of trunk  -T09.5 Injury of unspecified muscle and tendon of trunk  -T11.2 Dislocation, sprain and strain of unspecified joint and ligament of upper limb, level unspecified  -T11.5 Injury of unspecified muscle and tendon of upper limb, unspecified  -T13.2 Dislocation, sprain and strain of unspecified joint and ligament of lower limb, level unspecified  -T13.5 Injury of unspecified muscle and tendon of lower limb, unspecified  -T14.6 Injury of muscles and tendons of unspecified body region |
| Intracranial/  internal injuries | -S06.0~9 Intracranial injury  -S26.0~9 Injury of heart  -S27.0~9 Injury of other and unspecified intrathoracic organs  -S36.0~9 Injury of intra-abdominal organs  -S37.0~9 Injury of urinary and pelvic organs  -T06.5 Injuries of intrathoracic organs with intra-abdominal and pelvic organs  -S05.0~9 Injury of eye and orbit |
| Open wounds | -S01.0~9 Open wound of head  -S08.0~9 Traumatic amputation of part of head  -S11.0~9 Open wound of neck  -S18 Traumatic amputation at neck level  -S21.0~9 Open wound of thorax  -S28.0 Traumatic amputation of thorax  -S31.0~8 Open wound of abdomen, lower back and pelvis  -S38.2~3 Crushing injury and traumatic amputation of part of abdomen, lower back and pelvis  -S41.0~8 Open wound of shoulder and upper arm  -S48.0~9 Traumatic amputation of shoulder and upper arm level  -S51.0~9 Open wound of forearm  -S58.0~9 Traumatic amputation of forearm  -S61.0~9 Open wound of wrist and hand  -S68.0 Traumatic amputation of wrist and hand  -S71.0~8 Open wound of hip and thigh  -S78.0~9 Traumatic amputation of hip and thigh  -S81.0~9 Open wound of lower leg  -S88.0~9 Traumatic amputation of lower leg  -S91.0~7 Open wound of ankle and foot  -S98.0~4 Traumatic amputation of ankle and foot  -T01.0~9 Open wounds involving multiple body regions  -T05.0~9 Traumatic amputations involving multiple body regions  -T09.1 Open wound of trunk, level unspecified  -T09.6 Traumatic amputation of trunk, level unspecified  -T11.1 Open wound of upper limb, level unspecified  -T11.6 Traumatic amputation of upper limb, unspecified  -T13.1 Open wound of lower limb, level unspecified  -T13.6 Traumatic amputation of lower limb, unspecified  -T14.1 Open wound of unspecified body region |
| Injury to blood vessels | -S09.0 Injury of blood vessels of head, not elsewhere classified  -S15.0~9 Injury of blood vessels at neck level  -S25.0~9 Injury of blood vessels of thorax  -S35.0~9 Injury of blood vessels at abdomen, lower back and pelvis level  -S45.0~9 Injury of blood vessels at shoulder and upper arm level  -S55.0~9 Injury of blood vessels at forearm level  -S65.0~9 Injury of blood vessels at wrist and hand level  -S75.0~9 Injury of blood vessels at hip and thigh level  -S85.0~9 Injury of blood vessels at lower leg level  -S95.0~9 Injury of blood vessesl at ankle and foot level  -T06.3 Injuries of blood vessels involving multiple body regions  -T11.4 Injury of unspecified blood vessel of upper limb, level unspecified  -T13.4 Injury of unspecified blood vessel of lower limb, level unspecified  -T14.5 Inury of blood vessel(s) of unspecified body region |
| Superficial injuries,  contusions | -S00.0~9 Superificial injury of head  -S09.2 Traumatic rupture of ear drum  -S10.0~9 Superficial injury of neck  -S20.0~8 Superficial injury of thorax  -S30.0~9 Superficial injury of abdomen, lower back and pelvis  -S40.0~9 Superficial injury of shoulder and upper arm  -S50.0~9 Superficial injury of forearm  -S60.0~9 Superficial injury of wrist and hand  -S70.0~9 Superficial injury of hip and thigh  -S80.0~9 Superficial injury of lower leg  -S90.0~9 Superficial injury of ankle and foot  -T00.0~9 Superficial injuries involving multiple body regions  -T09.0 Superficial injury of trunk, level unspecified  -T11.0 Superficial injury of upper limb, level unspecified  -T13.0 Superficial injury of lower limb, level unspecified  -T14.0 Superficial injury of unspecified body region |
| Crushing injuries | -S07.0~9 Crushing injury of head  -S17.0~9 Crushing injury of neck  -S28.0 Crushing injury of thorax and traumatic amputation of part of thorax  -S38.0~1 Crushing injury and traumatic amputation of part of abdomen, lower back and pelvis  -S47 Crushing injury of shoulder and upper arm level  -S57.0~9 Crushing injury of forearm  -S67.0~8 Crushing injury of wrist and hand  -S77.0~2 Crushing injury of hip and thigh  -S87.0~8 Crushing injury of lower leg  -S97.0~8 Crushing injury of ankle and foot  -T04.0~9 Crushing injuries involving mulitple body regions  -T14.7 Crushing injury and traumatic amputation of unspecified body region |
| Burns | T20-T32 Burns and corrosions |
| Injury to nerves and  spinal cord | -S04.0~9 Injury of cranial nerves  -S14.0~6 Injury of nerves and spinal cord at neck level  -S24.0~6 Injury of nerves and spinal cord at thorax level  -S34.0~8 Injury of nerves and lumbar spinal cord at abdomen, lower back and pelvis level  -S44.0~9 Injury of nerves at shoulder and upper arm level  -S54.0~9 Injury of nerves at forearm level  -S64.0~9 Injury of nerves at wrist and hand level  -S74.0~9 Injury of nerves at hip and thigh level  -S84.0~9 Injury of nerves at lower leg level  -S94.0~9 Injury of nerves at ankle and foot level  -T06.0 Injuries of brain and cranial nerves with injuries of nerves and spinal cord at neck level  -T06.1 Injuries of nerves and spinal cord involving other multiple body regions  -T06.2 Injuries of nerves involving multiple body regions  -T09.3 Injury of spinal cord, level unspecified  -T09.4 Injury of unspecified nerve, spinal nerve root and plexus of trunk  -T11.3 Injury of unspecified nerve of upper limb, level unspecified  -T13.3 Injury of unspecified nerve of lower limb, level unspecified  -T14.4 Inury of nerve(s) of unspecified body region |
| Poisoning | -T36~T50 Poisoning by drugs, medicaments and biological substances  -T51~T65 Toxic effecs of substances chiefly nondicinal as to source |
| Other specified and unspecifiec injuries | -S09.7 Multiple injuries of head  -S09.8 Other specified injuries of head  -S09.9 Unspecified injury of head  -S19 Other and unspecified injuries of neck  -S29.0~9 Other and unspecified injuries of thorax  -S39 Other and unspecified injuries of abdomen, lower back and pelvis  -S49.0~9 Other and unspecified injuries of shoulder and upper arm  -S59.7~9 Other and unspecified injuries of forearm  -S69.7~9 Other and unspecified injuries of wrist and hand  -S79.7~9 Other and unspecified injuries of hip and thigh  -S89.7~9 Other and unspecified injuries of lower leg  -T06.8 Other specified injuries involving multiple body regions  -T07 Unspecified multiple injuries  -T09.8 Other specified injuries of trunk, level unspecified  -T09.9 Unspecified injury of trunk, level unspecified  -T11.8 Other specified injuries of upper limb, level unspecified  -T11.9 Unspecified injury of upper limb, level unspecified  -T13.8 Other specified injuries of lower limb, level unspecified  -T13.9 Unspecified injury of lower limb, level unspecified  -T14.8 Other injuries of unspecified body region  -T14.9 Injury, unspecified |

**Supplement Table 2.** ICD-10 codes for comorbid psychiatric disorder

| Comorbid psychiatric disorder | |
| --- | --- |
| Mood disorder | F30, F31, F32, F33, F34, F38, F39 |
| Anxiety disorder | F40,F41 |
| Stress-related disorder | F42 |
| Substance-related disorder | F10, F11, F12, F13, F14, F15, F16, F17, F18, F19 |
